# Excess short-term mortality in noncritical patients with atrial fibrillation presenting to the emergency department

**DOI:** 10.1007/s00508-021-01895-y

**Published:** 2021-06-21

**Authors:** Jan Niederdöckl, Michael Schwameis, Harald Herkner, Hans Domanovits

**Affiliations:** Department of Emergency Medicine, Medical University of Vienna, Währinger Gürtel 18–20/6D, 1090 Vienna, Austria

**Keywords:** Atrial fibrillation, Mortality, Risk prediction, Emergency medicine, Arrhythmia

## Abstract

**Background:**

Mortality data of non-critically ill patients presenting with symptomatic atrial fibrillation to an emergency department are scarce. We aimed to analyze the short-term mortality of these patients compared with that of the general Austrian population.

**Design/methods:**

This study analyzed a consecutive series of non-critically ill adults presenting to the emergency department at the Medical University of Vienna between 2012 and 2016 with complaints related to atrial fibrillation. The study outcome was mortality during the observation period. Age-specific and sex-specific mortality rates per 100 person-years were calculated and compared with the mortality rates of the Austrian population during the same period.

**Results:**

In total, 1754 patients with atrial fibrillation (43.1% female) were included in the study. During a median follow-up of 25 months, 248 of these patients died. Observed mortality rates were 7.8 per 100 person-years for females (95% confidence interval, CI 6.6–9.5) and 5.9 per 100 person-years for males (95% CI 5.0–7.1). Age-adjusted and sex-adjusted mortality rates were 2.8 (95% CI 2.3–3.3) and 2.7 (95% CI 2.2–3.2) per 100 person-years, respectively. Mortality rates for the Austrian population were 1.1 per 100 person-years for both females and males. Corresponding standardized mortality ratios were 2.5 for females (95% CI 2.1–3.0) and 2.4 for males (95% CI 2.0–2.9).

**Conclusion:**

The short-term mortality of patients with symptomatic atrial fibrillation presenting to the emergency department was substantially higher compared with the general Austrian population.

**Supplementary Information:**

The online version of this article (10.1007/s00508-021-01895-y) contains supplementary material, which is available to authorized users.

## Introduction

Identifying vulnerable, high-risk patients and specific subgroups is crucial for personalized medicine [1]. Patients with atrial fibrillation (AF) represent an opaque, heterogeneous, and extraordinarily fast-growing risk group [2, 3]. Risk management is difficult because this population varies widely in epidemiology, etiology, and comorbidities [2]. Mortality due to AF is already high in the absence of comorbidities. A widely cited study including a broad AF population reported a twofold increased risk of all-cause mortality in females and a 1.5-fold increased risk in males [4]. Whether these results also apply to subgroups, such as patients with symptomatic AF in the emergency department (ED) is unknown. Because the ED commonly serves as the first point of contact with the healthcare system, it plays a key role in the integrated management of AF patients [4]. As a cause of admission in 3–10% of all cases, AF is very common in this setting [5, 6]. In addition to acute treatment, the referral to further management must also be ensured here [4]. Because acute AF is rarely life-threatening in the ED, the vulnerability of these patients may not be fully recognized. Furthermore, original research on the mortality of patients with symptomatic AF in the ED is lacking. Therefore, the present study sought to evaluate the relative mortality of patients presenting to the ED with AF symptoms compared to the general Austrian population.

## Patients, materials, and methods

This single-center cohort study included a consecutive series of noncritical adults at least 18 years of age who presented with AF to the ED of the Medical University of Vienna between 2012 and 2016. This ED is part of the Vienna General Hospital, a tertiary care facility with approximately 83,000 ED visits annually. Around 600 of these visits (0.7%) are due to AF. All patients admitted and treated primarily due to AF and related complaints who gave informed consent were eligible for analysis. Critically ill patients with AF who were admitted and treated primarily for other reasons (including acute decompensated heart failure, stroke, acute coronary syndrome, sepsis and respiratory failure) were excluded from the study. Demographic data, comorbidities, medications, as well as onset and nature of symptoms were collected through patient interviews and medical record review.

The study outcome was mortality during the observation period. Mortality data were obtained from the Austrian death registry. Data on the Austrian population including mortality rates were obtained from the national central statistical office (Statistik Austria, Guglgasse 13, A‑1110 Vienna).

This study (https://clinicaltrials.gov/ study ID NCT03272620) was approved by the Ethics Committee of the Medical University of Vienna (approval number1568/2014) and conducted in accordance with the ethical principles of the Declaration of Helsinki (as amended at the 56th WMA General Assembly, Tokyo, Japan, 2008) and ICH GCP guidelines (1996).

### Statistical methods

We present continuous data as mean with standard deviation (SD), categorical data as absolute count with relative frequency (%). To test for differences in mortality, we used Student’s t‑test for continuous data. We calculated mortality rates between 2012 and 2016 from our patient sample using data from the Austrian death registry. The length of the observation period was the time between the date of first admission to the study center due to AF to the date of death or end of follow-up. We categorized patients into decades of age and merged the groups at both the upper and lower extremes to achieve appropriate group sizes. We calculated age-specific and sex-specific mortality rates and compared them to that of the Austrian population using official death statistics (Statistik Austria). We calculated standardized mortality ratios with 95% confidence intervals (CI) using indirect standardization [7]. We assumed that missing data were missing at random. Given the sample size, we decided not to use any methods for data imputation or replacement. We used MS Excel and Stata 14 for Mac (Stata Corp, College Station, TX, USA) for data management and analysis. Generally, a two-sided *p*-value less than 0.05 was considered to be statistically significant.

## Results

A total of 1754 non-critically ill patients with symptomatic AF treated between 2012 and 2016 in the ED of the Medical University of Vienna were analyzed (Supplemental Table 1). The cumulative observation time was 3654.7 patients/person-years with a median follow-up of 25.00 months (IQR 0.04–59.98 months). During follow-up, 248 patients died. Those who died tended to be older and female. Cardiovascular risk factors were more prevalent in patients who died.

**Fig. 1:**
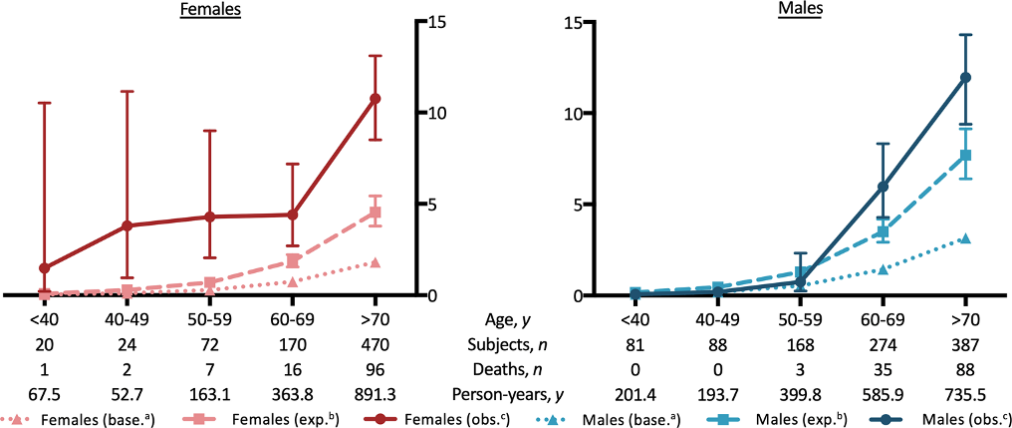
Mortality rates (*y-axes*) per 100 person-years by age in females and males. ^a^ Baseline mortality rate of the Austrian population, ^b^ expected age-adjusted and sex-adjusted mortality rate, ^c^ observed mortality rate of patients with AF presenting to the ED

During the observation period, the mortality rates of both female (7.8 per 100 person-years, 95% CI: 6.6–9.5) and male (5.9 per 100 person-years, 95% CI: 5.0–7.1) patients with symptomatic AF presenting to the ED was significantly higher than the mortality rate of the general Austrian population (1.1 for both sexes per 100 person-years; *p* < 0.001; Fig. 1). Age-adjusted and sex-adjusted mortality rates of AF patients were 2.8 (95% CI 2.3–3.3) and 2.7 (95% CI 2.2–3.2) per 100 person-years, respectively. This corresponded to standardized mortality ratios of 2.5 for females (95% CI 2.1–3.0) and 2.4 for males (95% CI 2.0–2.9;).

## Discussion

In the current era of precision medicine, simply knowing disease-associated complication rates is no longer sufficient [1] Identifying high-risk patients is crucial for sufficient management. In the current study, we were interested in non-critically ill patients with AF presenting to the ED. The increased mortality of these patients may not be intuitively obvious and may be less considered in everyday clinical practice. We found an at least 2.5-fold increased short-term mortality in adult patients presenting to the ED due to AF-related symptoms compared to the general Austrian population. Odutayo et al. recently confirmed the association between AF and all-cause mortality, showing a relative risk of 1.46 (95% CI: 1.39–1.54) based on a meta-analysis of 64 studies [8]. Based on the results of 8 studies [9–16], current European Society of Cardiology (ESC) AF guidelines report a 1.5-fold increased risk of all-cause death in males and a twofold increased risk in females [4]. Thus, the increased mortality of patients with AF is considered an accepted fact, with a mortality risk ranging from 1.5 to 2‑times higher than the unaffected population. Patients presenting to the ED substantially exceed those numbers.

These findings should be considered a red flag. The substantial effect size and the hard endpoint are highly suggestive to draw attention to the subject, especially because the explanation could be as simple as it is alarming: the need for improvements to the healthcare system. Interestingly, the study population had a significantly higher age-adjusted prevalence of important cardiovascular risk factors compared to the total Austrian AF population, regardless of survival. Arterial hypertension was present in 58.2% of this study cohort, compared to a prevalence of 21.6% in the Swedish study population of Andersson et al. [9]. This discrepancy may be partially explained by the higher prevalence of arterial hypertension (45.5%) among Austrians of the same age. Substantial differences were also observed for heart failure, diabetes mellitus, and COPD. The higher prevalence of risk factors in our population could explain the higher mortality. (supplementary Table 1) The idea of comorbidity playing the key role in the present phenomenon may also be supported by another fact: patients with comorbidities are known to have more severe AF-related symptoms [17, 18]. It is, therefore, possible that patients with cardiovascular risk factors and more severe AF-related symptoms [19] may be more likely to seek help at the ED rather than other medical facilities. Although the ED acts as an important primary contact point to the public health system [2, 4], its role is also important for further short-term and long-term treatment. If patients do not receive adequate subsequent care, they may end up in a vicious circle of recurrent ED visits with successful acute treatment but without sufficient follow-up. Thus, in these patients, structured outpatient management and regular check-ups should be guaranteed after acute treatment and discharge from an ED.

However, some essential questions need to be discussed: is the substantially higher mortality in patients seeking care in the ED due to AF symptoms because of a high rate of and/or insufficient treatment of comorbidities? Do AF patients with high cardiovascular risk who are treated at the ED obtain inadequate secondary care? If yes, then more extensive screening and more aggressive treatment should be considered, along with systematic improvement in subsequent referrals.

However, our findings may strengthen the awareness of the excess short-term mortality of non-critically ill AF patients presenting to an ED and may be a reminder of the importance of adequate follow-up care.

### Strengths/limitations

The greatest strength of our study is the robustness of the primary outcome measure, mortality, being assessed via the Austrian national death registry, providing information on all included patients; however, the study also has several limitations. First, the study included only a moderate number of patients with a short follow-up. Second, the national death registry provides limited information about the cause of death. Because there is no comprehensive information on the cardiovascular risk profile for the general Austrian population in the official death statistics, we could not adjust mortality rates for these risk factors. Another limitation concerns the comparison of AF patients and the general Austrian population, which includes a low proportion of people with AF; however, the statistics should be robust to this minor effect. Furthermore, we did not report the prevalence of structural heart disease as standardized echocardiography protocols have not yet been implemented in the diagnostic work-up of AF patients included in the AF registry at the ED. Further studies investigating causal inferences of our findings, however, will need to assess analyze and report structural heart changes as AF is often associated with underlying heart disease, which may increase the risk of mortality [20].

Finally, due to the single-center design, results will not necessarily apply to other settings or populations. Since unselected patients were consecutively enrolled our data reflect a real-world AF emergency cohort but further studies are necessary to confirm our study findings.

## Conclusion

Patients with symptomatic AF presenting to the ED have an excessive risk of short-term mortality and should receive structured outpatient management.

## Supplementary Information

**Supplemental Table 1** Characteristics of the general Austrian population and non-critically ill patients with symptomatic AF presenting to the emergency department; *MCI* myocardial infarction, *COPD* chronic obstructive pulmonary disease, *VKA* vitamin K antagonist, *NOAC* new oral anticoagulant, *N.A.* not available
