# Supplementary material for: Excess short-term mortality in noncritical patients with atrial fibrillation presenting to the emergency department
Source: Wien Klin Wochenschr. 2021 Jun 21;133(15-16):802–5. doi: 10.1007/s00508-021-01895-y (PMC8373724; doi:10.1007/s00508-021-01895-y)
Supplement: Supplementary file 1 — Supplemental Table 1 Characteristics of the general Austrian population and non-critically ill patients with symptomatic AF presenting to the emergency department; MCI myocardial infarction, COPD chronic obstructive pulmonary disease, VKA vitamin K antagonist, NOAC new oral anticoagulant, N.A. not available [file 508_2021_1895_MOESM1_ESM.docx]

**GENERAL AF PATIENTS -**

**POPULATION 2014 Emergency Department**

*n = 2 073 204 n = 1754*

**Clinical**

Age, years mean (SD) >60 67 (±14.9)

Female gender, % (n) 56 43 (756)

CHA2DS2-VASc mean (SD) *N.A.*  2 (±1.7)

**Comorbidities**

Heart failure, % (n) 11.3 21.9 (385)

Hypertension, % (n) 45.9 58.2 (1020)

Diabetes mellitus, % (n) 11.9 16.5 (289)

Stroke, % (n) 2.1 5.5 (19)

Ischemic heart disease, % (n) 8.5 18.1 (318)

Previous MCI, % (n) *N.A.* 9.1 (159)

Peripheral artery disease, % (n) *N.A.* 4.1 (72)

Hyperlipidemia, % (n) *N.A.* 25.8 (452)

COPD, % (n) 8.4 9.7 (171)

**Medication**

Beta-blockers, % (n) *N.A.* 37.9 (665)

Amiodaron, % (n) *N.A.* 15.7 (279)

VKA, % (n) *N.A.* 21.7 (380)

NOAC, % (n) *N.A.* 8.7 (152)

Diuretics, % (n) *N.A.* 20.9 (366)

**Mortality rate (per 100 pry)**

Female 1.1 7.8 (6.6-9.5)

Male 1.1 5.9 (5.0-7.1)

**Supplemental Table 1** Characteristics of the Austrian general population and of AF patients presenting to the emergency department ; Abbreviations*:* Myocardial Infarction (MCI), Chronic Obstructive Pulmonary Disease (COPD), Vitamin K Antagonist (VKA), New Oral Anticoagulant (NOAC), Not Available (N.A.), patients/person-years (pry).
